# Signals of sustainability transition: Sensing enabling factors through cultural initiatives

**DOI:** 10.1186/s40410-022-00187-w

**Published:** 2023-02-11

**Authors:** Grazia Concilio, Irene Bianchi, Ilaria Tosoni

**Affiliations:** DASTU Politecnico di Milano, Via Bonardi 3, 20133 Milan, Italy

**Keywords:** Urban sustainability transition, Cultural initiatives, Social impacts, Initiative-based learning, Scaling mechanisms

## Abstract

This article reports a first attempt to combine the analysis of socio-technical mechanisms and initiative-based learning to sense sustainability transition signals in cities. Relying on the analysis of cultural initiatives in six European Cities, the study identifies those factors that enabled social impact generation. It then formulates hypotheses about their contribution to the inception and rooting of sustainability transition dynamics. As a result, the article proposes a set of analytical categories of enabling factors acting across niches and regimes. The same factors are then reinterpreted by referring to scaling mechanisms (*scaling up*, *out* and *deep*). The proposed analytical scheme seeks to offer a broader reflection on the conceptual and methodological challenges related to sensing and interpreting urban sustainability transition pathways.

## Introduction

Urban literature increasingly affirmed the need for systemic perspectives to disentangle transformative trajectories affecting cities. Among the many conceptual frameworks attentive to the complexity of social, spatial and ecological (urban) phenomena, those adopting a socio-technical transition approach (Geels 2002, 2005, 2011; Geels and Schot 2007; Grin et al. 2010) gained particular momentum. Their innovative contribution is grounded in modelling systemic change in terms of mutual interactions of processes across the three levels of socio-technical systems: *niches* (as “incubation rooms for radical innovations”, see Geels 2005: 450), *regimes* (i.e. “the deep structure that accounts for the stability of an existing socio-technical system”, see Geels 2004: 27) and *landscapes* (i.e. long-term, exogenous trends which form “gradients for action from which it is hard to deviate”, Geels 2005: 451). The tremendous explanatory potential of this perspective has been evidenced by the myriad of works focussing on sustainability transition processes (Ernst et al. 2016; Geels 2018), concerned with social innovation and with “a phase-change in which new actors, relationships, logics, norms and performance criteria will emerge” (Turnheim et al. 2015: 241).

Nevertheless, the detection and understanding of sustainability transition processes *in the making* still pose significant analytical challenges (Turnheim et al. 2015; Pekkarinen and Melkas 2019). Difficulties are encountered in positioning the “observation point” to capture transition dynamics that emerge and develop in complex and open systems, have multiple drivers and nonlinear causalities, and affect interdependent systems’ components at multiple levels. Other challenges relate to incorporating the temporal and spatial dimensions (see Hodson and Marvin 2010; Raven et al. 2012). Still, others refer to reading sustainability transition signals in their interplay with other processes of social transformation, which co-evolve through more or less purposive actions by multiple agents.

This article responds to the call for comprehensive analytical schemes integrating different approaches, e.g. quantitative modelling, open-ended exploration of interactions across system levels, and context-sensitive analysis of initiative-based transformations (Turnheim et al. 2015). While recognising that the mere observation of specific actions and their impacts does not allow to infer the existence and direction of transition pathways, the article reflects on the detection of “transition signals”. To this end, it explores the potential of combining the observation of specific (cultural) initiatives and the analysis of transition pathways through “scaling mechanisms” (Moore et al. 2015; Riddell and Moore 2015). Particular attention is paid to patterns of interaction between local niches and regimes^1^ (Geels and Schot 2007) at the urban scale and their potential contribution to the generation of transformative effects (Concilio et al. 2019). In the development of the analytical framework, the study focuses on cultural initiatives implemented at the local level, in recognition of their transformative potential in urban settings (e.g., Sacco and Tavano Blessi 2009; James 2014; Sacco et al. 2014) towards collective well-being, better urban environments and social cohesion (European Commission 2018).

In sum, this article reports the work done to develop an analytical model for sensing sustainability transition signals with a particular focus on cultural initiatives and their transformative power through social impact generation. The article combines conceptual with empirical work. After clarifying why culture-driven social impacts are relevant to exploring sustainability transition processes, it discusses shortcomings in conceptualising and analysing the “culture—sustainability transition” binomial in the urban sphere. The paper then proposes and illustrates a taxonomy of enabling factors affecting social impact generation and formulates hypotheses about their potential significance as signals for unfolding systemic change in urban contexts. Foundation of the reflection is a qualitative study on culture-led projects and policies developed in 6 European cities and conducted under the umbrella of the Horizon 2020 MESOC Project.^2^ Lastly, enabling factors are discussed in relation to “scaling mechanisms” (Moore et al. 2015; van den Bosh and Rotmans 2008) in order to substantiate their potential relevance as sensors of progress on transition pathways.

## Theoretical and analytical coordinates

Cities are networked systems rich in emerging and potentially disruptive social, spatial and digital practices, experiments, and initiatives (Evans et al. 2016), which gain transformative capacity from mutual interactions and cross-fertilisation (Concilio et al. 2019). Cities work as social laboratories, nesting innovation niches into a collective learning system that supports social value production (Gutzmer 2016). This constant process of collective experience and learning gains even more relevance in directing innovation experiments, practices and, in the end, policies towards sustainability transition (Ernst et al. 2016; Geels 2018; Loorbach, in this issue). As global sustainability is critically dependent on urban sustainability (Bugliarello 2006; Walsh et al. 2006), the focus and understanding of urban systems and their social and physical change dynamics are essential to support the needed paradigm shift.

Urban sustainability transition necessarily asks for new approaches integrating the social dimension in shaping “positive futures” for nature and people (Rana et al. 2020; UNDP et al. 2021; Loorbach 2021). From a sustainability transition perspective, leveraging cultural actions is key as it allows to work on two levels: experiment and short-term social impacts generation on one side and long-term learning and systemic change on the other. In line with local development theories, the first level recognises the driving role of local cultural, human and social capital and indicates cultural action as essential for the quality of life and cohesiveness of local communities (Sacco and Tavano Blessi 2009: 1131). As for the second level, the role of culture and cultural initiatives can be traced back to changes in dominant logics, paradigms, values, individual behaviours, ways of value production and exchange, and, therefore, modes of social interaction and organisation (Loorbach 2022, in this issue; Pelling and Manuel-Navarrete 2011). Beyond academia, international institutions increasingly acknowledge the transformative potential of culture as well. The New European Agenda for Culture (EC 2018), for example, identifies three main areas in which the contribution of cultural initiatives has proven to be effective in generating significant social impacts and—ultimately—transformative effects: social cohesion, urban regeneration, and well-being.

Despite this attention, the relation between culture, urban transformation, and systemic change has often been neglected. Also, a deeper understanding of factors contributing to social impact generation towards sustainability transition is lacking. In addition, the assessment of cultural policies’ contribution to urban and social change is mostly economy-oriented and often overlooks the dark side of policies based on the so-called creative industry (Scott 2006; Florida 2002), such as gentrification, social isolation, and inequality. In methodological terms, the contribution of culture and cultural initiatives to long-term transformations is still mostly limited to quantitative assessment, which underestimates intangible and not quantifiable social impacts. Moreover, assessment models mainly focus on specific impact domains, not capturing the cross-cutting social and cultural consequences of public or private actions (Vanclay 2002: 190; Cicerchia 2021). Detecting the contribution to systemic change toward a more sustainable and inclusive society is a challenging task—beyond the already high complexity of social impact assessment (Clifford 2014).

The study illustrated in this article considers that the increasing take up in many European cities of cultural initiatives aiming at synergic social impacts relevant in a sustainability transition perspective can be interpreted as a signal of ongoing transition-oriented transformations. It observes how cultural initiatives targeting social impact generation emerge at both regime and niche levels, as well as at their interface, often activating processes of cumulative change and re-organization proper of “scaling mechanisms” (Moore et al. 2015), which explain how new structures (e.g. organizational settings) may emerge from novel practices through a sequence of located experimental and learning activities in a variety of contexts (van den Bosh and Rotmans 2008; Riddell and Moore 2015):

- *Scaling deep* includes shifts in ways of thinking, organising, and acting. This mechanism includes changes in values and perspectives, habits and routines, as well as physical, institutional or economic structures. Two modes of *scaling deep* emerge in the literature (Smith and Raven 2012; van den Bosh and Rotmans 2008; Riddell and Moore 2015): (i) niche-driven, e.g. through the insurgency of niches, challenges towards the regime, the exploration and discovery of values through experiments; (ii) top-down as well as collaborative conditions of the interaction between niches and regime.
- *Scaling out* implies replicating novel practices and related experiments in different contexts, as well as linking values, practices and structures to other functions or domains. Through this mechanism, different attempts towards the adoption of novel practices exist simultaneously and build on each other over time.
- Finally, *scaling up* is defined as embedding a novel practice in dominant ways of thinking (value), doing (practices) and organising (structure), at the level of a societal system.

Adopting scaling mechanisms as overall descriptors of the processes at play, the work tries to capture those factors specifically enabling the generation of social impacts, and so contributing to sustainability transition.

## Methodological notes

This study drafts an analytical scheme to capture sustainability transition signals. In doing so, it considers sustainability transitions as “never-ending social processes of progressive social change”, involving a progressive shift in “needs, wants, institutions, culture and practices” (Kemp et al. 2007: 1) at multiple levels. Also, the article recognises that “transitions are complex processes that cannot be overseen or steered from one viewpoint [… as] they are emergent outcomes of interactions between social groups with myopic views and differing interests, strategies and resources” (Geels 2005: 453). As transition pathways cannot be observed in their entirety, nor can they be inferred through the bare observation of single initiatives, the article makes the hypothesis that factors enabling the generation of social impacts may work as sensors to detect signals of ongoing transition-oriented transformation. The article works on two interconnected analytical dimensions: (i) cultural initiatives targeting social impacts and (ii) their contribution to the activation and consolidation of sustainability transition dynamics. At the intersection of these two dimensions, factors enabling social impact generation are identified as potential “sensors” to qualify sustainability transition signals and, therefore, as the main constituent of the analytical scheme.

This article focuses on cultural initiatives targeting three specific—yet interconnected—social impacts domains: well-being, urban regeneration, and social cohesion. In line with the New European Agenda for Culture (EC 2018), those social spheres are identified as crucial *loci* for the emergence and consolidation of culture-led transformative dynamics in cities. Since specific technical conditions for a quantitative and systematic assessment of impacts and their determinants are not always present in the field of culture, alternative methods for the evaluation, appreciation, and description of impact-generating phenomena are emerging (Cicerchia 2021). The proposed analytical scheme considers an approach based on storytelling to extract from stories the types of information needed to detect regularities and recurrences able to provide a consistent description of the phenomena according to a defined framework (Davies and Dart 2005). Accordingly, the study unfolds through the following analytical phases:

1. Preliminary identification of relevant impact determinants, according to literature, as per their relation to transformative dynamics and scaling processes.
2. Collection and analysis of 18 cultural initiatives from 6 European cities (see Table 4 in Annex). The initiatives have been investigated through desk work combined with a qualitative survey.
3. Textual analysis of the case study reports, aimed at identifying—in the promoters’ perception and narration—clues of the relation between specific actions, choices, contextual and external conditions and the obtained impact. These actions are defined as “case-specific enabling factors” (*Level I*) and referred to the general impact determinants presented in literature.
4. Generalization of case-specific enabling factors through a clustering exercise, working through proximity and similarity across recurrent semantics and described situations. This “comprehensive enabling factors” (*Level II*) are then interpreted as sensors of sustainability transition in the making: signals of an ongoing shift towards sustainability in the definition of urban policies leveraging cultural actions as measures to ensure critical impact capacity.

**Fig. 1:**
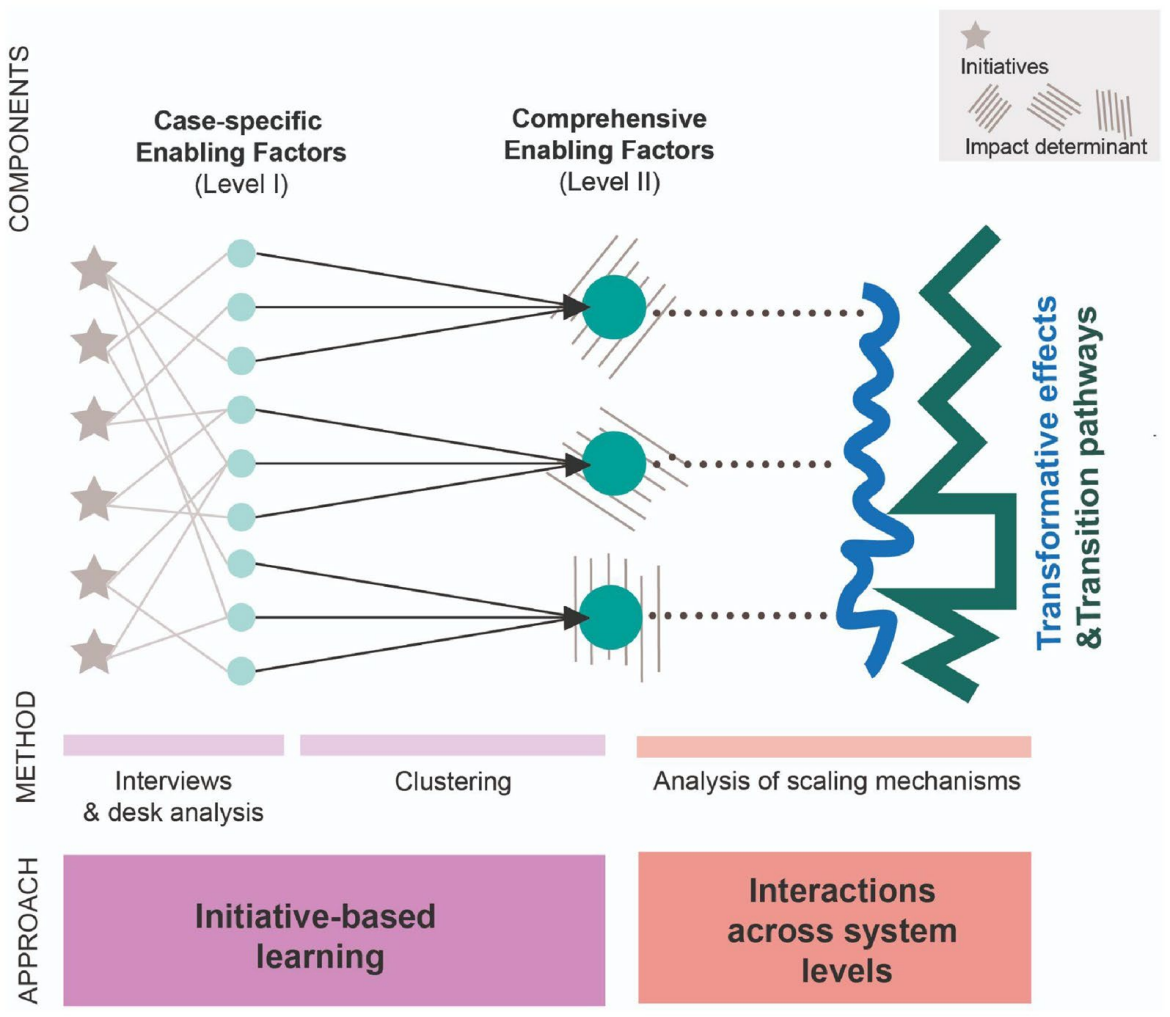
Components of the analytical scheme

The components of the analytical schemes and their relation are represented in Fig. 1.

1. *Preliminary identification of impact determinants*
  The authors have identified five main families of impact determinants in the literature.
  *Networks and partnerships,* related to social capital relations affecting the emergence, development and implementation of a (cultural) action (Caniëls and Romijn 2008; López-García et al. 2019).
  *Resources and infrastructures,* in terms of access to (and mobilisation of) resources of different nature: monetary (Mulgan et al. 2011) as well as material, cognitive and spatial.
  *Norms and regulations*, which can reinforce, spread, and diffuse the adoption of certain practices and approaches able to strengthen the effectiveness and transformative power of policies and initiatives (Moore 2017).
  *Narrative and discourses*, i.e., elements of the local socio-cultural environment which can influence the process or become components of the action design itself (Smith 1999; Allon and Sofoulis 2006; Komendantova and Neumueller 2020).
  *Knowledge and abilities*, in terms of access to key information or knowledge as well as previous experience, which can constitute the key input to activate a change process or to design a specific action (Frantzeskaki and Rok 2018); agents with specific characteristics, motivations, and goals can drive transformative processes at different scales.
  As literature emphasises, these areas affect values (e.g. shared beliefs and perspectives), structures (particularly in terms of institutional, organisational and regulatory settings) and possibly practices (e.g. behaviours, routines and spontaneous actions) and thus have an impact on problem structuring, envisioning and long-term goals formulation, as well as processes of agenda building, negotiating, networking and coalition building (Kemp et al. 2007; Rotmans and Loorbach 2009).
2. *Collection and analysis of cultural initiatives*
  The choice fell on initiatives explicitly targeting MESOC Project’s social impact domains, i.e. well-being, urban regeneration and social cohesion. The analysis of 18 cultural initiatives from 6 European cities (see Annex)—namely Milan (IT), Issy-Les-Moulineaux (FR), Valencia (ES), Cluj-Napoca (RO), Barcelona (ES) and Athens (GR)—has been carried out. The selected projects include both innovative and well-established cultural initiatives openly targeting the generation of social value in the impact domains. The cultural initiatives identified are promoted or supported by a variety of urban actors: local administrations, citizens groups and associations, or cultural organisations and enterprises (e.g. Foundations and Museums). They include cultural policies and strategies, private and public cultural initiatives, culture-related community practices, as well as sectoral measures using cultural activities in the design and implementation phases. Also, these initiatives pertain to different cultural domains, including heritage, visual arts, performing arts, audio-visual and multimedia, architecture and art crafts (following the categorisation proposed by EUROSTAT’s ESSnet-Culture report—EC 2012). Being heterogeneous in terms of typology, scope and scale, the selected cultural initiatives provide a good sample of the complex processes and interactions informing cultural production and its effect on different aspects of urban life. Cultural initiatives have been analysed through a triangulation of qualitative research methods, including desk search, questionnaires, semi-structured interviews with key actors (policy makers and cultural operators), and analysis of grey literature. Overall, the analysis looked at the whole cycle through which cultural initiatives have been designed, planned and implemented. The investigation follows a process-focused approach (Langley et al. 2013). A case study report has been completed for each cultural initiative.
3. *Identification of case-specific enabling factors*
  The 18 case study reports were analysed through a textual analysis, which allowed the identification of relevant and possibly recurrent factors enabling, in the actors’ perspective and storytelling, the generation of social value. A preliminary set of factors has been developed through a simple process of text extraction and cleaning from the materials provided by the participants (transcript of answers to the questionnaire, documents and reports, answers to interviews). The cleaning operation concerned merely anonymization, reference to specific locations and projects, harmonisation of the syntactic structure of the sentences. The list of factors can be found in Table 1, Column 1.
4. *Definition of comprehensive enabling factors*
  **Table 1:** Case-specific and comprehensive enabling factors

Case-specific enabling factors (Level I)	Comprehensive enabling factors (Level II)	General impact determinants
• Support of local, regional, national and European cultural institutions • Political recognition from public institutions at multiple levels • Broad support from different typologies of institutional stakeholders	Political recognition and support	Networks and partnerships
• Cross-sectoral coordination within local public institutions • Emergence or consolidation of private–public partnerships • Definition of multi-level institutional agreements • Establishing collaborations with schools and local educational bodies • Establishing collaborations with healthcare institutes • Establishing collaboration with international cultural institutions • Establishing collaboration with research networks • Connecting to existing international networks	Emergence and consolidation of collaborative schemes	
• Involvement of local cultural operators • Involvement of early-career artists • Involvement of healthcare practitioners • Active engagement of neighbourhood organisations • Active engagement of vulnerable target groups	Involvement of co-beneficiaries	
• Endorsement by renowned experts or professionals • Active engagement of renowned experts or professionals	Involvement of renowned experts or professionals	
• Activation of synergies with initiatives from the same context • Activation of synergies with similar initiatives from different contexts	Synergies with other initiatives	
• Access to European or National funds • Allocation of dedicated budget from local or regional authorities	Access to financial resources	Resources and infrastructures
• Participation to national and international calls for funding • Definition of public–private sponsorship agreements • Launch of crowd-funding campaigns • Activation of broad support networks • Definition of a diversified funding strategy	Mobilisation of financial resources	
• Use of available public venues • Use of available private venues • (Temporary) activation of underused spaces • Availability of dedicated equipment • Adoption of new technological infrastructures	Access to and activation of spaces and infrastructures	
• Activation of pre-existing working groups • Activation of a new dedicated working group • Activation of professionals and practitioners from the impact domain • Involvement of a core group of engaged citizens • Involvement of volunteers	Activation of dedicated working groups	
• Benefit from favourable local policy frameworks • Benefit from enabling norms and regulation from the cultural sector • Benefit from favourable sectoral norms and regulations from the impact domain • Alignment with existing policies and planning documents • Benefit from national policies supporting local cultural venues • Activation of synergies with urban rebranding or regeneration strategies	Building on enabling norms and regulations	Norms and regulations
• Contribute to the modification of existing norms and regulations • Introduce innovations in decision-making processes • Inclusion as “best practice” in policy documents • Participation in the co-design of policy recommendations • Introduce new priorities in the policy agenda • Contribute to the cross-sectoral alignment of policy documents • Contribute to the development of new services • Contribute to the formalisation of cultural practices in regulatory and strategic documents	Rooting in norms and regulations from the impact domains	
• Deep rooting of cultural activities in neighbourhood dynamics • Definition of a context-sensitive cultural offer • Efforts to achieve the trust of target populations • Adoption of words, images, and symbols from local communities • Use of words, images, and symbols that refer to local identity and dynamics • Embedment of multiple points of view	Establishing synergies with local dynamics and identity	Narratives and discourses
• Use of words, images, and symbols to strategically align with well-established narratives from the cultural sector • Use of words, images, and symbols to strategically align with well-established narratives from the impact domain • Adaptation of high-level principles to context-specific dynamics	Aligning with dominant policy narratives	
• Establishment of synergies with broader urban rebranding strategies • Adoption of a holistic approach to healthcare • Prioritisation of mental health after COVID-19 • Promotion of active ageing lifestyle • Adoption of a welcoming and well-being focused design	Aligning with discourses emerging in the impact domain	
• Development of an identity as a new reference point for target communities • Emphasis on the contribution to new urban functions • Emphasis on the contribution to social innovation • Pioneering in the use of digital technologies	Emphasising innovation and novelty	
• Capitalise on the image and reputation of renowned experts or professionals	Capitalising on the image of guest stars	
• Foster the emergence of new discourses in the local context • Foster the emergence of new discourses in the impact domain	Fostering the emergence of new discourses	
• Involve experts and practitioners from the impact domain • Involve cultural operators • Embed local and experiential knowledge in the cultural activities	Building on the skills and expertise of involved stakeholders	Knowledge and abilities
• Target skill development of vulnerable social groups • Target skill development of healthcare practitioners • Target skill development of early-career artists • Target skill development of participants	Enhancing skills and expertise
  To generalize a set of comprehensive enabling factors, the sentences previously identified have been clustered through a process of semantic association. Clusters have then been labelled and categorised according to thematic criteria providing an effective synthesis and description of the contents. Lastly, the identified comprehensive enabling factors have been discussed in light of their potential link to scaling mechanisms, to indirectly debate their contribution to sustainability transition. Steps 3 and 4 have been cyclically repeated by using the identified enabling factors to capture additional actions from the case studies and, vice versa, a more specific list of actions has been used to enrich and specify the list of enabling factors.

## Sensing contribution to sustainability transition: a first attempt

### Towards the analytical schemes

Through the application of the methodological process described above, the different components have been specified as illustrated in Table 1. The first column shows case-specific enabling factors identified from the analyses of the selected cultural initiatives. In the development of the analytical scheme, they play a functional role, not presuming to be a complete list. The second column shows the results of the clustering process, identifying comprehensive enabling factors with a higher generalisation degree. For example, while the relevance of “broad support from different types of institutional stakeholders” (Column 1) has been detected in a most of the cases analysed, the associated comprehensive enabling factor “political recognition and support” (Column 2) might work as a lens for sensing transformative effects, to be applied by analysts across different contexts.

### Illustrative example

*To what extent are comprehensive enabling factors adequate to sense sustainability transition signals?* In our assumptions, their adequacy depends on their capacity to sense transformative effects. As a preliminary test, the following examples illustrate how comprehensive enabling factors can orient initiative-based learning and support the identification of key mechanisms across system levels, ultimately allowing the formulation of hypothesis about ongoing transformative effects (see Tables 2 and 3).

**Table 2:** Illustrative example: scaling up

General impact determinants	Comprehensive enabling factors	Initiative-based learning	Interactions across system levels	Transformative effects *Resulting from scaling-up dynamics*
		Case-specific enabling factors *Resulting through the observation of one/more initiatives in one context*	Identification of key scaling mechanisms	
Knowledge and abilities	Enhancing skills and expertise	• Experts from the impact domain (e.g. urban regeneration) have been included in the development of cultural actions • Local knowledge has been embedded in the design of cultural activities	**Scaling up** Cultural actions at the niche level start growing and becoming more stable. In the long-term, this trend might affect institutional structures, e.g. through re-alignment or innovation **Key interaction**: Collaboration between niches and regimes	• Emergent practices formalised through norms and regulation • New pervasive narratives created • New values consolidated • Widened adoption of novel practices from social impact domains • Expanded adoption of novel practices by synergising functions and domains • Learning
	Facilitating knowledge acquisition and diffusion	• Collaboration agreements have been signed with local research bodies • Results from the cultural actions are disseminated throughout local networks		
Norms and regulations	Rooting in norms and regulations from the impact domains	• The Involvement of cultural operators in urban regeneration processes is formally recognized • Cultural operators participate in co-design experiments seeking to support policy recommendations		
Narratives and discourses	Emphasising innovation and novelty	• Innovative cultural actions become a reference point for the development of neighbourhood initiatives		
Resources and infrastructures	Access to financial resources	• Private–public partnerships are signed • Sponsorship agreements are promoted by cultural institutions		
	Access and activation of spaces and infrastructures	• Underused cultural venues are activated • New technological infrastructures are developed		
Networks and partnerships	Political recognition and support	• The Municipality formally acknowledges the relevance of the initiatives launched		
	Emergence and consolidation of collaborative schemes	• The Cultural Unit and the Urban Planning Department of the Municipality develop new forms of collaborations • A new network involving local cultural associations and institutions emerges

**Table 3:** Illustrative example: scaling deep

General impact determinants	Comprehensive enabling factors	Initiative-based learning	Interactions across system levels	Transformative effects *Resulting from scaling-deep dynamics*
		Case-specific enabling factors *Resulting through the observation of one/more initiatives in one context*	Identification of key scaling mechanisms	
Knowledge and abilities	Facilitating experience exchange	• Local fora development to ease experience exchange among practitioners working in urban regeneration • Feedback about ongoing activities is exchanged within neighbourhood associations	**Scaling deep** Creation/birth of constellations of practices implementing learning experiments (mainly *regime driven*) **Key interactions**: Regime drives, supports, enables niches; niches activate experiments with very low involvement of regime; niches challenge the regime	• Local rooting of new practices and related values • Local learning through experiments on influencing culture, practices and structures • Widened constellation ecosystem • Local learning through experiments on influencing culture, practices and structures • New culture, practices and structures’ potential shown to regimes
	Supporting shared reflection	• Citizens involved in the development of the initiative(s) discuss the importance of cultural actions in relation to local identity issues • Citizens involved in the development of the initiative(s) discuss about their expectations for the future of the neighbourhood		
Norms and regulations	Rooting in norms and regulations from the impact domains	• Cultural operators participate in co-design experiments seeking to support policy making		
Narratives and discourses	Aligning with discourses emerging in the impact domain	• In line with well-being-oriented approaches to urban planning, cultural operators launch activities to promote active lifestyle		
Resources and infrastructures	Activation of dedicated working groups	• Practitioners from the cultural sector collaborate with local institutions		
	Access and activation of spaces and infrastructures	• Underused urban spaces are activated • Private venues are made available for public exhibitions		
Networks and partnerships	Emergence and consolidation of collaborative schemes	• Cultural operators collaborate with local schools for the re-activation of neighbourhood spaces		
	Involvement of co-beneficiaries	• Culture-led projects targeting e.g., urban regeneration directly involve vulnerable target groups		

An illustrative exercise helps clarifying how “comprehensive enabling factors” (Table 1, Column 2) can ease the identification of factors affecting social impact generation in specific contexts. The presence and combination of multiple comprehensive enabling factors can reveal transition dynamics, which are then detected and described through scaling mechanisms (Tables 2 and 3, Column 4). The exercise allows enquiring different scaling mechanisms at play, and reflecting on the capacity of constellations of initiatives to (i) produce change in local contexts, e.g. “supporting transformative learning or communities of practice” (*scaling deep*, Riddell and Moore 2015: 4); (ii) produce change in organisational settings, e.g. through the modification of policies or institutional routines (*scaling up*); and (iii) diffuse practices across contexts and domains (*scaling out*). As the latter would require a cross-contextual perspective that is out of the scopes of this article, Tables 2 and 3 show a first attempt to map comprehensive enabling factors onto scaling up and scaling deep mechanisms, and to formulate hypotheses about transformative effect.

The cultural initiatives that provided input to this article have been selected at a local level and mostly refer to “institutional” cultural policies or well-established cultural niche-experiments. In terms of scaling, we mostly observe *scaling up* mechanism concerning the availability, accessibility, or mobilisation of key resources, as well as interactions with normative and policy settings (see Table 2). The observation of these mechanisms, combined with initiative-based learning, can reveal if and how (cultural) niches are growing and becoming more stable, so that they can either “substitute” the regime (e.g., by becoming service providers) or influence its action (e.g., by informing institutional or normative change). Accordingly, the interpretative framework can support the observation of institutional change, which develops through gradual hybridization of functions and integration of competences; as a consequence, new organizational settings become standard. For example, as far as culture-led sustainable urban regeneration is concerned, in most cases culture is not responsible for the mere supply of cultural infrastructures. Cultural initiatives are also able to work as social aggregators, to drive behavioural change, to enhance relational capital. In other words, they can either take over some of the “regime functions” in the impact domain (e.g. in relation to urban design and place-making) or force it to share power. It is nonetheless in the mechanisms of *scaling deep* that signals of systemic change can be observed in their full amplitude (Geels 2004). The development of constellations of practices implementing and building on learning experiments (either niche-pushed or regime-pulled) signals a stabilization of knowledge, skills and ways of operating which affects the quality and agency of networks and partnerships as well as their capacity to leverage on their knowledge to gain regime support and commitment. Recurring semantics and narratives indicate how this capability of producing and broadening knowledge translates into values and perspectives, which are gradually embedded in contexts both in terms of practices (behaviours and ways of doing things) and discourses (story-telling, political discourse).

## Concluding remarks

This article reports on a first attempt to merge a socio-technical perspective and initiative-based learning into an analytical framework aimed at sensing signals of sustainability transition. Building on the analysis of selected cultural initiatives, this paper proposes a set of analytical categories to disentangle dynamic factors able to affect social impact generation, thus contributing to the creation of social value. Furthermore, it identifies interpretative categories based on scaling mechanisms (scaling up, scaling out and scaling deep), which are adopted to observe the potential transformative effects of those factors and describe the level and quality of niche–regime interactions. The proposed scheme allows for a preliminary identification of factors that affect sustainability transition in its initial stages, and contribute to the consolidation of transition-oriented practices in (institutional and organisational) structures and norms.

The identified comprehensive enabling factors seem to be promising in sensing ongoing transition-oriented transformations. As illustrated in the previous section, they can help capturing transformative effects taking place through different scaling mechanisms. Notably, sensing complex transformative dynamics requires sensing multiple enabling factors acting, at times, individually or, mostly, synergically, as in the cases of local consolidation of new practices and embedded values; learning through experiments redefining cultural meanings, practices and structures; widening the niche–regime constellations ecosystems; generating protective spaces (Smith and Raven 2012) for niche-experiments; stabilizing practices through norms and regulations. The reliability of enabling factors as sensors of ongoing sustainability transition processes still needs validation and testing, but the provided analytical scheme has the potential to effectively enable the detection of actions having transitional quality.

Although it considers a limited number of examples (18 cultural actions in 6 European cities), the study provides interesting insights concerning the sensing of interactions between niches and regimes. Also, the categories identified allow the formulation of some hypotheses on key aspects of ongoing sustainability transition pathways. Moreover, the study provides insights into social impact analysis, introducing a broader, process-focused perspective, attentive to the interpretation of complex transformative phenomena. The adopted analytical approach indeed allows to read social impact generation dynamics in their interaction with socio-technical transition mechanisms; therefore, it has the potential to overcome reductionist and deterministic biases connected to output-based evaluation models.

Nevertheless, the attempt to sense and interpret sustainability transition signals through cultural initiatives presents a number of limitations. First, the study has been developed in the framework of a European Project, and it is affected by its problem setting and conditions. Outcomes are therefore at this stage still partial and conditioned by the project’s aims, in particular regarding cases selection and numerousness. A second limitation concerns the positive bias of the selected cultural actions, which only include well-established initiatives seeking to generate social impact. If on the one side the focus on “positive” examples allows reflecting on dynamics and contextual pre-conditions that are relevant for the generation of social impacts, on the other it does not sense issues of dissent and contestation, nor it detects conflicts and hurdles that are inherently embedded in experimentation, and that are not indifferent to the nature of innovation (Köhler et al. 2019). While the authors are aware of this analytical bias, they still consider the framework valuable for providing relevant—yet partial—insights into micro-transformation emerging at the local level.

Further research is needed to overcome these limitations. On the one hand, enlarging the number and variety of cultural actions would allow the identification of new dynamics affecting social impact generation. On the other hand, a broader sample of cases would allow a refining of the analytical categories to include conflictual and disruptive dynamics that might affect transition dynamics. Finally, with a greater variety of cultural initiatives it would be possible to further test the effectiveness of scaling mechanisms as a mean to read regime–niches interactions.

At the same time, the analysis of social impact generation dynamics could be broadened to foster a context-aware understanding of the processes taking place according to four potential different analytical clustering criteria: the cultural action/impact relation; the urban dynamics through a collection of cases from the same context; the dynamics related to specific social domains (health and wellbeing, etc.); the analysis of their contribution to enabling or rooting transformation processes associated with transition dynamics (detectable at both the urban ecosystem and macro-scale). At the same time, the analytical framework could be broadened and applied to capture differences in the attitude of the agents involved (e.g. in terms of awareness, resourcefulness, willingness to experiment), thus opening new reflections on their role in the making of sustainability transition.

## Data Availability

Not applicable.

## Annex

See Table 4.

**Table 4:** Short description of the cultural initiatives

City and main social impact domain	Initiative	Short description
Cluj-Napoca (RO) *Health and well-being*	Unspeakable	Publicly funded workshop carried out by Cluj Cultural Centre in collaboration with composer D.P. Cohen and Notes & Ties organisation. The project offers young people the opportunity to express themselves through music. The project is inspired by UK research showing that artistic expression can support well-being and be part of treatment for mild to moderate mental illness
	TABLO	European Project seeking to develop an e-learning training course to integrate art therapy into the daily routines of healthcare personnel working with long-term patients. The package applies to people with various conditions and across many different cultural settings, it promotes art in therapeutic environments and supports a holistic approach to well-being
	Inner space	Project exploring the contribution of art and cultural participation to the well-being of the city’s inhabitants. It develops a Culture and Well-being Think Tank, hosts an annual international forum, and pilots design and artistic interventions. The project seeks to support the inclusion of psychological well-being as a strategic factor for the sustainable development of Cluj-Napoca, making it a reference point on the topic
Valencia (ES) *Health and well-being*	Caixa dels Records—Memorias de una vida	Project by the Ethnographic Museum of Valencia (ETNO) under the umbrella of the “Museums-Museus Inclusius programme”, promoted by the Municipal Council. Through reminiscence group therapy, the project seeks to support people with Alzheimer’s disease or other dementias in their initial stage and elderly with cognitive deficits associated with normal ageing
	Museus per la Salut—Records de Festa al Museu Faller de València	This project, addressed to early-stage Alzheimer patients, uses cultural resources as inputs to stimulate their long-term memory through reminiscence therapy. To do so, it favours access to culture for the target group, usually excluded from mainstream cultural offers
	Ocio Inclusivo y Sensibilización	Non-profit Project seeking to help people with autism improve their personal development through music and other arts. It is run by a team of professionals specialised in Music Therapy and Music Education, and it targets people with functional intellectual diversity. This inclusive leisure project promotes initiatives to support the social integration of people with autism and their relatives
Athens (EL) *Citizens’ participation and empowerment*	Culture in the Neighbourhood	Initiative promoted by the Athens Municipality and its cultural network (Athens Culture Net). It consists of interactive events organised in several cultural venues and open public spaces seeking to activate local cultural spaces and resources and to enhance cultural participation of residents and visitors (children, young, seniors, professionals) in their neighbourhoods
	Athens Garden Festival	Festival organised since 2012 by Athens Art Network, in collaboration with Athens Municipality and with local culture, youth, and sports organisations It hosts urban acoustic, visual and music performances in parks and gardens (most of the time at Athens National Gardens) and at the same time promotes sustainability educational activities
	Athens Escape Route	Digital treasure hunt guiding players in a virtual exploration of Athens and its culture. By solving puzzles, questions and knowledge quizzes, the gamers get in contact with Athens cultural organisations, neighbourhoods, history, architecture, street art, hidden secrets and unknown attractions
Barcelona (ES) *Citizens’ participation and empowerment*	Apropa Cultura	Network of cultural programmes seeking to support access to culture for people at risk of social exclusion or with disabilities. Over 60 theatres, concert halls, festivals and museums across Catalonia and the Balearic Islands adhere to the initiative
	En Palabras—Relatos migrantes	Barcelona-based creative writing collective made up of people from Latin America interested in researching, writing and sharing stories about the life experience of migration. It proposes creative and literary exploration workshops guided by professional poets and writers, in which migrants and refugees share their experiences and try to build communitarian documentation and new narratives on Latin American migrations in Spain
	Xamfrà	Socio-educational Centre using the arts to guarantee the right of access to cultural and artistic practice for all citizens. It follows the objectives of the Fundació L’ARC Musica, which revolve around principles of equality/equity, recognition of diversity, positive interaction, diverse participation and feelings of belonging
Milan (IT) *Urban regeneration*	IL PERTINI	Cultural centre and a public library based in Cinisello Balsamo (Milan). This public-led project, which involved private and third sector stakeholders, redesigned the public library and the surrounding area, imagining the library as a multi-functional space to host workshops and cultural initiatives
	CASCINA MARTESANA	Multifunctional cultural space based in a former industrial brownfield. The project promotes cultural initiatives, including concerts, performances, exhibitions, and events tackling wellness and body care, environmental sustainability, social cohesion. Its purpose is to enhance the social engagement of citizens in a broader process of cultural and urban regeneration
	Caravansaray Selinunte San Siro	NGO-led artistic and social project, supported by the Municipality of Milan. Five authors living in the peripheral neighbourhood of San Siro have created a series of dialogues with the residents, collecting stories, life experiences and short narrative ideas. These materials were used to write a theatre pièce, brought back to the neighbourhood through short theatrical incursions
Issy-Les-Moulineaux (FR) *Urban regeneration*	Le Temps des Cerises	Project developed in the framework of the first SMART neighbourhood in France. Located in an ancient military fort, transformed into a multifunctional cultural space, it hosts artistic and digital activities and meetings for all audiences. The building, open to everyone, is managed by CLAVIM, an association of the City of Issy-les-Moulineaux, specialized in culture and youth
	Musée Français de la Carte à Jouer	Cultural space promoting place-specific cultural initiatives targeting different social groups. The Pavillon des Conti hosts the Gallery of the History of Issy-les-Moulineaux, which illustrates the city’s evolution from a social, urban and industrial perspective
	Espace Andrée Chedid	Open to all family configurations, the Espace combines a parent–child place, a day-care centre, a parent-baby unit, reception and listening places for teenagers, structures dedicated to child-parent-grandchildren relationships. It proposes activities in the fields of digital technology, culture, prevention and health. It pays particular attention to social support to families and vulnerable social groups

## Footnotes

1 In the context of this article, “regimes” include all private and public institutions, rules, problem agendas, guiding principles, standards and decisions affecting urban phenomena and their cultural dimension.

2 www.mesoc-project.eu.
